# Supplementary material for: Sus1 maintains a normal lifespan through regulation of TREX-2 complex-mediated mRNA export
Source: Aging (Albany NY). 2022 Jun 29;14(12):4990–5012. doi: 10.18632/aging.204146 (PMC9271307; doi:10.18632/aging.204146)
Supplement: Supplementary Figures [file aging-14-204146-s001.pdf]

SUPPLEMENTARY FIGURES

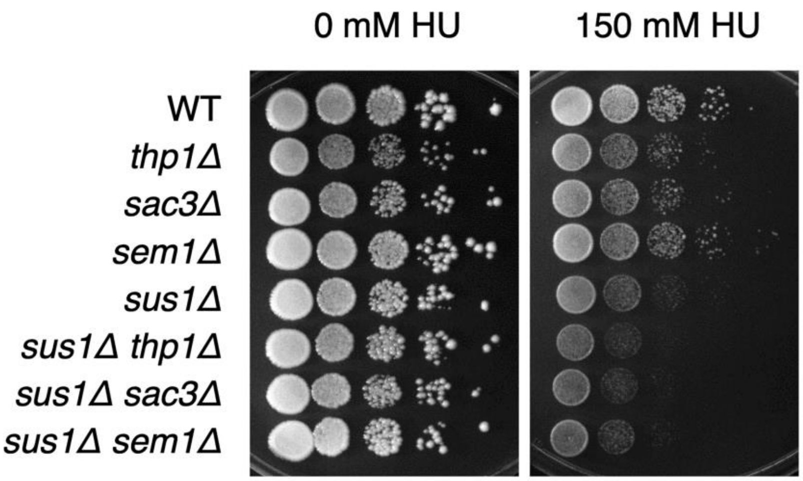

Supplementary Figure 1. HU sensitivity assays in double deletion *sus1Δ* strains with TREX-2 mutants. The indicated mutants were spotted onto YPD plates with or without 150 mM HU, as described in Figure 1E.

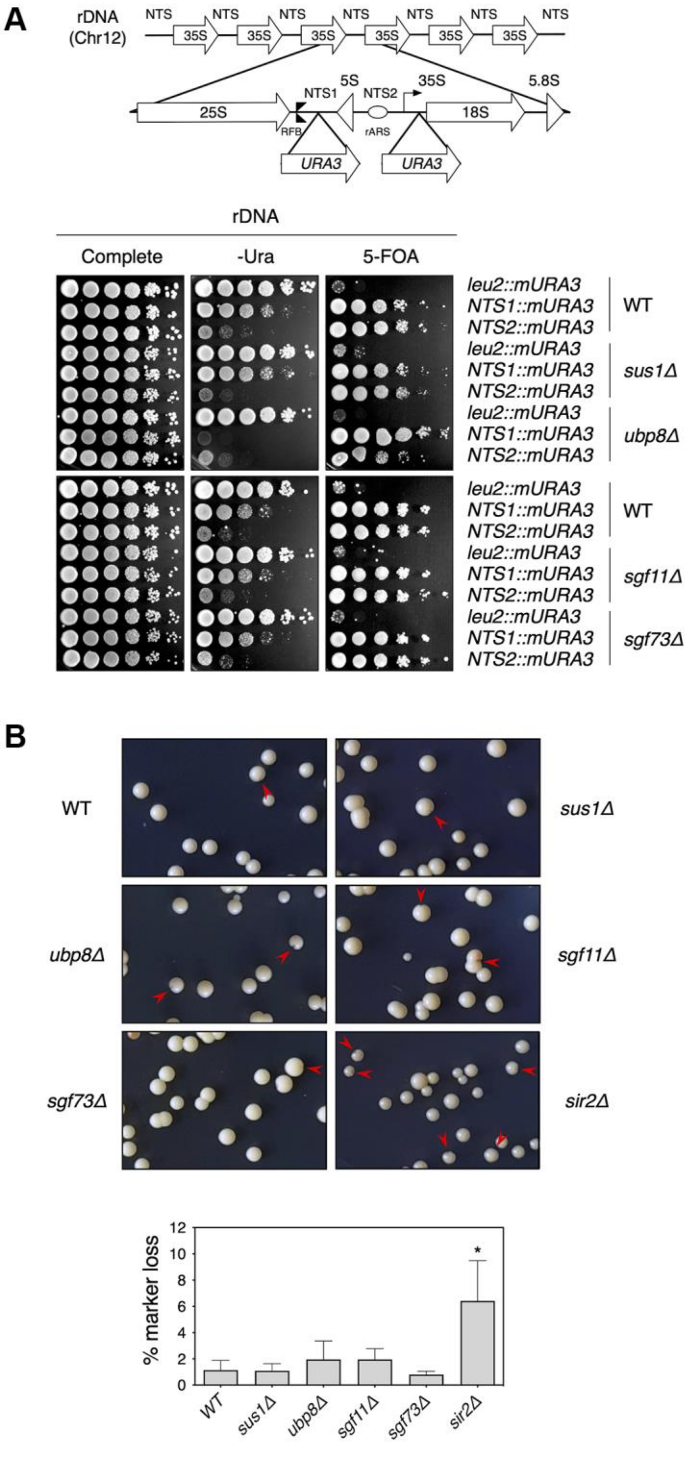

Supplementary Figure 2. *SUS1* deletion does not affect rDNA silencing and recombination. (**A**) Schematic diagram (top) showing an rDNA unit embedded within a tandem array on chromosome XII with the position of *mURA3* reporters inserted into NTS1 or NTS2. The 35S pre-rRNA encoding the 18S, 5.8S, and 25S rRNAs is separated by NTS1 and NTS2. The locations of RFB (double triangle), ARS replication origin (oval), 5S rRNA gene (triangle), and 35S transcription start site (bent arrow) are represented. *URA3*-based rDNA silencing assays (bottom) were carried out in DMY2798 (*leu2::mURA3*), DMY2804 (*RDN1-NTS1::mURA3*), or DMY2800 (*RDN1-NTS2::mURA3*) strains with the indicated deletions. (**B**) The frequency of unequal rDNA crossovers was monitored by loss of the *ADE2* gene located within the rDNA array for the WT (W303R) strain and the indicated deletion strains. Pictures of plates are shown in the top panels. The percentage of *ADE2* gene loss (% marker loss) was calculated as the ratio of red-sectored colonies to the total number of colonies and is shown in the bottom panel. Completely red colonies were excluded. Error bars indicate the SD from two repetitions, and asterisks indicate statistically significant differences between the mutant and WT strains (ns, not significant; $^{*}P < 0.05$).

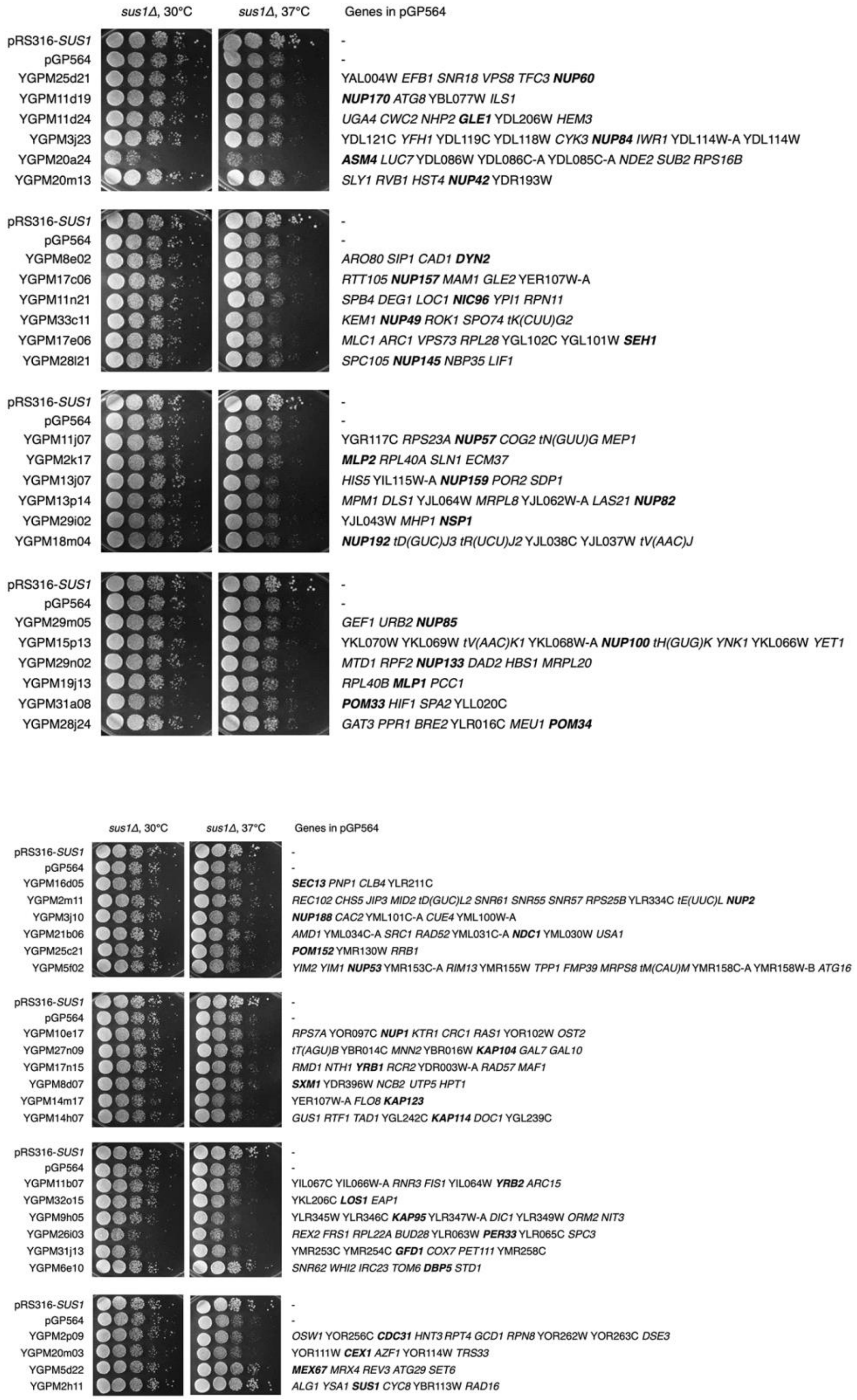

Supplementary Figure 3. Screening of NPC-related genes for the suppression of growth defects in *sus1Δ* cells. Growth analysis of *sus1Δ* strains, including the indicated plasmids, as described in Figure 1E. Genes on the plasmids (pGP564) are listed on the right of each panel.

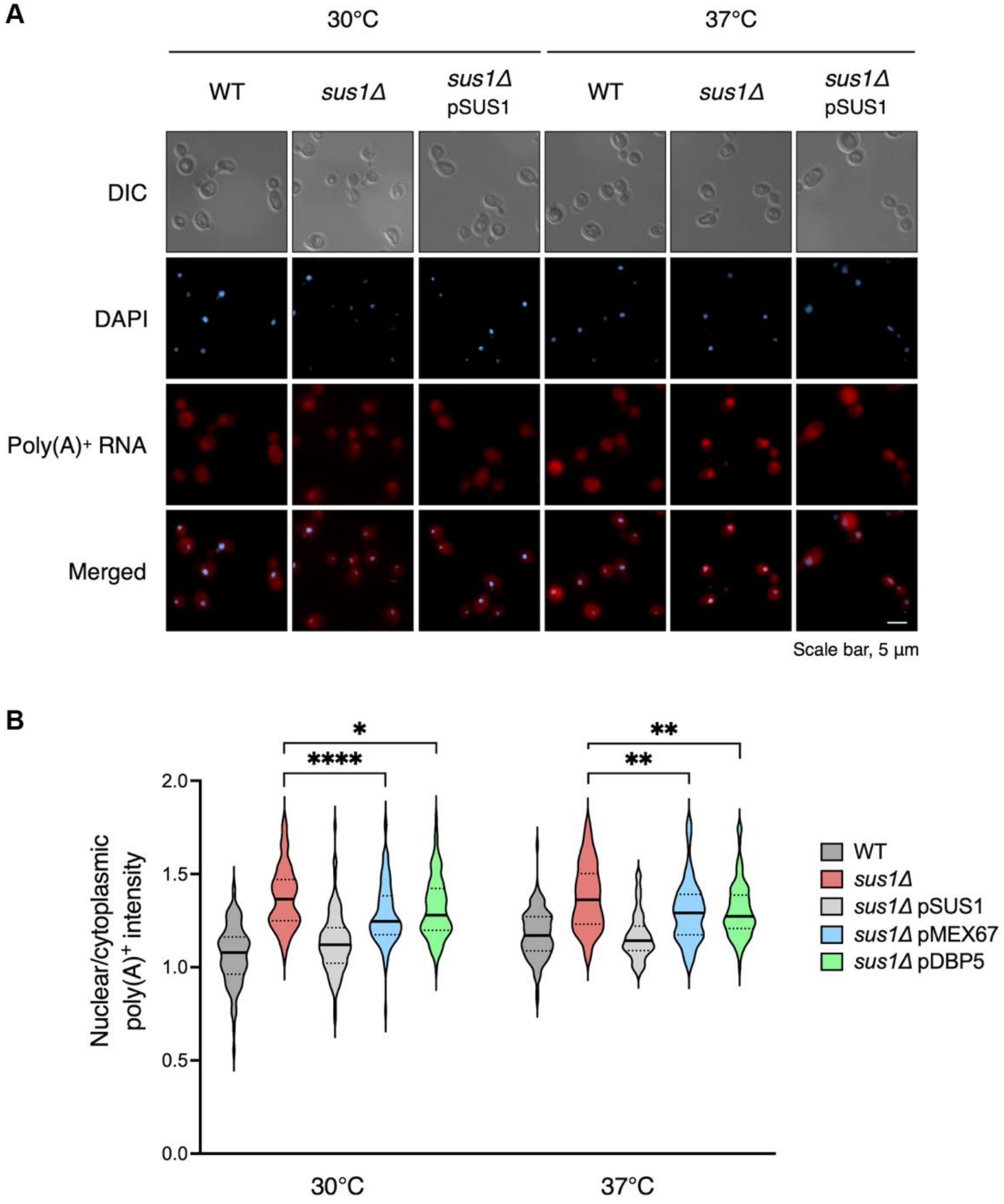

Supplementary Figure 4. The mRNA export defect induced by *sus1Δ* was observed at both 30°C and 37°C. (**A**) Poly(A)$^+$ RNA FISH analysis of the WT, *sus1Δ*, and *sus1Δ* containing pRS316-SUS1 strains, as described in Figure 5A. (**B**) Violin plot of poly(A)$^+$ RNA FISH results for the strains used in Figure 5A at both 30°C and 37°C. The medians and quartiles are marked as thick and dotted lines, respectively. $^{****}P < 0.0001$; $^{**}P < 0.01$; $^{*}P < 0.05$ (Student's *t*-test between the indicated pairs of values).

A

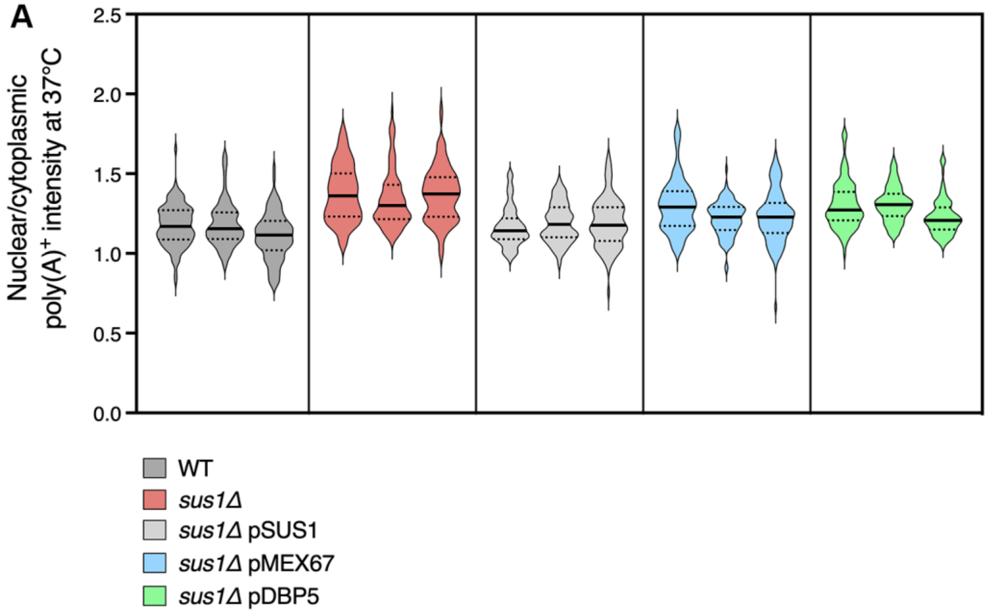

B

Experiments		Cell numbers	Mean (Nuclear/cytoplasmic poly(A)+ intensity at 37°C)
1	WT	103	1.176154
	sus1Δ	112	1.376510
	sus1Δ pSUS1	100	1.167284
	sus1Δ pMEX67	104	1.294548
	sus1Δ pDBP5	103	1.304344
2	WT	102	1.182704
	sus1Δ	105	1.337783
	sus1Δ pSUS1	105	1.188931
	sus1Δ pMEX67	103	1.218946
	sus1Δ pDBP5	104	1.309805
3	WT	103	1.107127
	sus1Δ	102	1.369916
	sus1Δ pSUS1	102	1.192707
	sus1Δ pMEX67	110	1.231505
	sus1Δ pDBP5	104	1.229178

Supplementary Figure 5. Additional copies of *MEX67* or *DBP5* rescued the mRNA export defect in *sus1Δ* cells. (**A**) Violin plot of poly(A)+ RNA FISH results presented in Figure 5A, as described in Supplementary Figure 4B. The nuclear/cytoplasmic poly(A)+ intensity of each replicate is plotted. (**B**) The cell numbers and mean nuclear/cytoplasmic poly(A)+ intensity ratio in (**A**) are shown.
