# Supplementary material for: Sus1 maintains a normal lifespan through regulation of TREX-2 complex-mediated mRNA export
Source: Aging (Albany NY). 2022 Jun 29;14(12):4990–5012. doi: 10.18632/aging.204146 (PMC9271307; doi:10.18632/aging.204146)
Supplement: Supplementary Table 1 [file aging-14-204146-s002.docx]

**Supplementary Table 1. Strains used in this study.**

| Strain | Genotype | Source |
| --- | --- | --- |
| BY4741 | *MATa ura3∆0 leu2∆0 his3∆1 met15∆0* | EUROSCARF |
| FY231 | *MATa ura3∆0 leu2∆0 his3∆1 met15∆0 ubp8∆::KanMX4* | EUROSCARF |
| FY390 | *MATa ura3∆0 leu2∆0 his3∆1 met15∆0 thp1∆::KanMX4* | EUROSCARF |
| FY391 | *MATa ura3∆0 leu2∆0 his3∆1 met15∆0 sac3∆::KanMX4* | EUROSCARF |
| FY395 | *MATa ura3∆0 leu2∆0 his3∆1 met15∆0 cyh2 mex67::KanMX4(DAmP)* | Open Biosystems |
| FY396 | *MATa ura3∆0 leu2∆0 his3∆1 met15∆0 cyh2 mtr2::KanMX4(DAmP)* | Open Biosystems |
| FY399 | *MATa ura3∆0 leu2∆0 his3∆1 met15∆0 sgf11∆::KanMX4* | EUROSCARF |
| FY402 | *MATa ura3∆0 leu2∆0 his3∆1 met15∆0 sgf73∆::KanMX4* | EUROSCARF |
| FY432 (W303R) | *MATa ura3-1 leu2-3,112 trp1-1 his3-11,15 ade2-1 can1-100 RAD5+ RDN1::ADE2* | Won-Ki Huh & Leonard Guarente |
| FY433 (DMY2798) | *MATa ura3-1 leu2-3,112 trp1-1 his3-11,15 ade2-1 can1-100 leu2::mURA3* | Won-Ki Huh & Danesh Moazed |
| FY434 (DMY2804) | *MATa ura3-1 leu2-3,112 trp1-1 his3-11,15 ade2-1 can1-100 RDN1-NTS1::mURA3* | Won-Ki Huh & Danesh Moazed |
| FY435 (DMY2800) | *MATa ura3-1 leu2-3,112 trp1-1 his3-11,15 ade2-1 can1-100 RDN1-NTS2::mURA3* | Won-Ki Huh & Danesh Moazed |
| FY451 | *MATa ura3∆0 leu2∆0 his3∆1 met15∆0 sem1∆::KanMX4* | EUROSCARF |
| FY739 | *MATa ura3∆0 leu2∆0 his3∆1 met15∆0 MEX67-GFP (S65T)::HIS3MX6* | Seong-Tae Kim & Won-Ki Huh |
| FY740 | *MATa ura3∆0 leu2∆0 his3∆1 met15∆0 DBP5-GFP (S65T)::HIS3MX6* | Seong-Tae Kim & Won-Ki Huh |
| SY022 | *MATa ura3∆0 leu2∆0 his3∆1 met15∆0* [pRS316] | This study |
| SY495 | *MATa ura3∆0 leu2∆0 his3∆1 met15∆0 sus1∆::HIS3MX6* | This study |
| SY529 | *MATa ura3∆0 leu2∆0 his3∆1 met15∆0 sir2∆::HIS3MX6* | This study |
| SY532 | *MATa ura3∆0 leu2∆0 his3∆1 met15∆0 sac3∆::KanMX4 sir2∆::HIS3MX6* | This study |
| SY551 | *MATa ura3-1 leu2-3,112 trp1-1 his3-11,15 ade2-1 can1-100 RAD5+ RDN1::ADE2 sir2∆::KanMX4* | [1] |
| SY556 | *MATa ura3∆0 leu2∆0 his3∆1 met15∆0 thp1∆::KanMX4 sir2∆::HIS3MX6* | This study |
| SY557 | *MATa ura3∆0 leu2∆0 his3∆1 met15∆0 ubp8∆::KanMX4 sir2∆::HIS3MX6* | This study |
| SY558 | *MATa ura3∆0 leu2∆0 his3∆1 met15∆0 sgf73∆::KanMX4 sir2∆::HIS3MX6* | This study |
| SY559 | *MATa ura3∆0 leu2∆0 his3∆1 met15∆0 sgf11∆::KanMX4 sir2∆::HIS3MX6* | This study |
| SY575 | *MATa ura3-1 leu2-3,112 trp1-1 his3-11,15 ade2-1 can1-100 leu2::mURA3 sus1∆::KanMX6* | This study |
| SY576 | *MATa ura3-1 leu2-3,112 trp1-1 his3-11,15 ade2-1 can1-100 leu2::mURA3 ubp8∆::KanMX4* | This study |
| SY577 | *MATa ura3-1 leu2-3,112 trp1-1 his3-11,15 ade2-1 can1-100 leu2::mURA3 sgf11∆::KanMX4* | This study |
| SY578 | *MATa ura3-1 leu2-3,112 trp1-1 his3-11,15 ade2-1 can1-100 leu2::mURA3 sgf73∆::KanMX4* | This study |
| SY580 | *MATa ura3-1 leu2-3,112 trp1-1 his3-11,15 ade2-1 can1-100 RDN1-NTS1::mURA3 sus1∆::KanMX6* | This study |
| SY581 | *MATa ura3-1 leu2-3,112 trp1-1 his3-11,15 ade2-1 can1-100 RDN1-NTS1::mURA3 ubp8∆::KanMX4* | This study |
| SY582 | *MATa ura3-1 leu2-3,112 trp1-1 his3-11,15 ade2-1 can1-100 RDN1-NTS1::mURA3 sgf11∆::KanMX4* | This study |
| SY583 | *MATa ura3-1 leu2-3,112 trp1-1 his3-11,15 ade2-1 can1-100 RDN1-NTS1::mURA3 sgf73∆::KanMX4* | This study |
| SY585 | *MATa ura3-1 leu2-3,112 trp1-1 his3-11,15 ade2-1 can1-100 RDN1-NTS2::mURA3 sus1∆::KanMX6* | This study |
| SY586 | *MATa ura3-1 leu2-3,112 trp1-1 his3-11,15 ade2-1 can1-100 RDN1-NTS2::mURA3 ubp8∆::KanMX4* | This study |
| SY587 | *MATa ura3-1 leu2-3,112 trp1-1 his3-11,15 ade2-1 can1-100 RDN1-NTS2::mURA3 sgf11∆::KanMX4* | This study |
| SY588 | *MATa ura3-1 leu2-3,112 trp1-1 his3-11,15 ade2-1 can1-100 RDN1-NTS2::mURA3 sgf73∆::KanMX4* | This study |
| SY652 | *MATa ura3∆0 leu2∆0 his3∆1 met15∆0 sus1∆::HIS3MX6* | This study |
| SY653 | *MATa ura3∆0 leu2∆0 his3∆1 met15∆0 sus1∆::KanMX6* | This study |
| SY654 | *MATa ura3∆0 leu2∆0 his3∆1 met15∆0 sus1∆::KanMX6* | This study |
| SY699 | *MATa ura3∆0 leu2∆0 his3∆1 met15∆0 sus1∆::KanMX6 sir2∆::HIS3MX6* | This study |
| SY784 | *MATa ura3∆0 leu2∆0 his3∆1 met15∆0 sus1∆::HIS3MX6 sac3∆::KanMX4* | This study |
| SY785 | *MATa ura3∆0 leu2∆0 his3∆1 met15∆0 sus1∆::HIS3MX6 sem1∆::KanMX4* | This study |
| SY786 | *MATa ura3∆0 leu2∆0 his3∆1 met15∆0 sus1∆::HIS3MX6 ubp8∆::KanMX4* | This study |
| SY787 | *MATa ura3∆0 leu2∆0 his3∆1 met15∆0 sus1∆::HIS3MX6 sgf11∆::KanMX4* | This study |
| SY794 | *MATa ura3∆0 leu2∆0 his3∆1 met15∆0 sem1∆::KanMX4* *sir2∆::HIS3MX6* | This study |
| SY797 | *MATa ura3∆0 leu2∆0 his3∆1 met15∆0 sus1∆::HIS3MX6 sgf73∆::KanMX4* | This study |
| SY825 | *MATa ura3-1 leu2-3,112 trp1-1 his3-11,15 ade2-1 can1-100 RAD5+ RDN1::ADE2 sgf11∆::KanMX4* | This study |
| SY826 | *MATa ura3-1 leu2-3,112 trp1-1 his3-11,15 ade2-1 can1-100 RAD5+ RDN1::ADE2 ubp8∆::KanMX4* | This study |
| SY827 | *MATa ura3-1 leu2-3,112 trp1-1 his3-11,15 ade2-1 can1-100 RAD5+ RDN1::ADE2 sgf73Δ::KanMX4* | This study |
| SY831 | *MATa ura3-1 leu2-3,112 trp1-1 his3-11,15 ade2-1 can1-100 RAD5+ RDN1::ADE2 sus1∆::HIS3MX6* | This study |
| SY955 | *MATa ura3∆0 leu2∆0 his3∆1 met15∆0 sus1∆::HIS3MX6* [pRS316-*SUS1*] | This study |
| SY971 | *MATa ura3∆0 leu2∆0 his3∆1 met15∆0 sus1∆::HIS3MX6* [pGP564] | This study |
| SY972 | *MATa ura3∆0 leu2∆0 his3∆1 met15∆0 sus1∆::HIS3MX6* [YGPM2h11] | This study |
| SY973 | *MATa ura3∆0 leu2∆0 his3∆1 met15∆0 sus1∆::HIS3MX6* [YGPM5d22] | This study |
| SY974 | *MATa ura3∆0 leu2∆0 his3∆1 met15∆0 sus1∆::HIS3MX6* [YGPM6e10] | This study |
| SY975 | *MATa ura3∆0 leu2∆0 his3∆1 met15∆0 sus1∆::HIS3MX6* [YGPM25a15] | This study |
| SY976 | *MATa ura3∆0 leu2∆0 his3∆1 met15∆0 sus1∆::HIS3MX6* [YGPM17i23] | This study |
| SY981 | *MATa ura3∆0 leu2∆0 his3∆1 met15∆0 sus1∆::HIS3MX6* [pRS316-*SUS1*] [pGP564] | This study |
| SY982 | *MATa ura3∆0 leu2∆0 his3∆1 met15∆0 sus1∆::HIS3MX6* [pRS316-*SUS1*] [YGPM25d21] | This study |
| SY983 | *MATa ura3∆0 leu2∆0 his3∆1 met15∆0 sus1∆::HIS3MX6* [pRS316-*SUS1*] [YGPM11d19] | This study |
| SY984 | *MATa ura3∆0 leu2∆0 his3∆1 met15∆0 sus1∆::HIS3MX6* [pRS316-*SUS1*] [YGPM11d24] | This study |
| SY985 | *MATa ura3∆0 leu2∆0 his3∆1 met15∆0 sus1∆::HIS3MX6* [pRS316-*SUS1*] [YGPM3j23] | This study |
| SY986 | *MATa ura3∆0 leu2∆0 his3∆1 met15∆0 sus1∆::HIS3MX6* [pRS316-*SUS1*] [YGPM20a24] | This study |
| SY987 | *MATa ura3∆0 leu2∆0 his3∆1 met15∆0 sus1∆::HIS3MX6* [pRS316-*SUS1*] [YGPM20m13] | This study |
| SY988 | *MATa ura3∆0 leu2∆0 his3∆1 met15∆0 sus1∆::HIS3MX6* [pRS316-*SUS1*] [YGPM8e02] | This study |
| SY989 | *MATa ura3∆0 leu2∆0 his3∆1 met15∆0 sus1∆::HIS3MX6* [pRS316-*SUS1*] [YGPM17c06] | This study |
| SY990 | *MATa ura3∆0 leu2∆0 his3∆1 met15∆0 sus1∆::HIS3MX6* [pRS316-*SUS1*] [YGPM11n21] | This study |
| SY991 | *MATa ura3∆0 leu2∆0 his3∆1 met15∆0 sus1∆::HIS3MX6* [pRS316-*SUS1*] [YGPM33c11] | This study |
| SY992 | *MATa ura3∆0 leu2∆0 his3∆1 met15∆0 sus1∆::HIS3MX6* [pRS316-*SUS1*] [YGPM17e06] | This study |
| SY993 | *MATa ura3∆0 leu2∆0 his3∆1 met15∆0 sus1∆::HIS3MX6* [pRS316-*SUS1*] [YGPM28l21] | This study |
| SY994 | *MATa ura3∆0 leu2∆0 his3∆1 met15∆0 sus1∆::HIS3MX6* [pRS316-*SUS1*] [YGPM11j07] | This study |
| SY995 | *MATa ura3∆0 leu2∆0 his3∆1 met15∆0 sus1∆::HIS3MX6* [pRS316-*SUS1*] [YGPM2k17] | This study |
| SY996 | *MATa ura3∆0 leu2∆0 his3∆1 met15∆0 sus1∆::HIS3MX6* [pRS316-*SUS1*] [YGPM13j07] | This study |
| SY997 | *MATa ura3∆0 leu2∆0 his3∆1 met15∆0 sus1∆::HIS3MX6* [pRS316-*SUS1*] [YGPM13p14] | This study |
| SY998 | *MATa ura3∆0 leu2∆0 his3∆1 met15∆0 sus1∆::HIS3MX6* [pRS316-*SUS1*] [YGPM29i02] | This study |
| SY999 | *MATa ura3∆0 leu2∆0 his3∆1 met15∆0 sus1∆::HIS3MX6* [pRS316-*SUS1*] [YGPM18m04] | This study |
| SY1000 | *MATa ura3∆0 leu2∆0 his3∆1 met15∆0 sus1∆::HIS3MX6* [pRS316-*SUS1*] [YGPM29m05] | This study |
| SY1001 | *MATa ura3∆0 leu2∆0 his3∆1 met15∆0 sus1∆::HIS3MX6* [pRS316-*SUS1*] [YGPM15p13] | This study |
| SY1002 | *MATa ura3∆0 leu2∆0 his3∆1 met15∆0 sus1∆::HIS3MX6* [pRS316-*SUS1*] [YGPM29n02] | This study |
| SY1003 | *MATa ura3∆0 leu2∆0 his3∆1 met15∆0 sus1∆::HIS3MX6* [pRS316-*SUS1*] [YGPM19j13] | This study |
| SY1004 | *MATa ura3∆0 leu2∆0 his3∆1 met15∆0 sus1∆::HIS3MX6* [pRS316-*SUS1*] [YGPM31a08] | This study |
| SY1005 | *MATa ura3∆0 leu2∆0 his3∆1 met15∆0 sus1∆::HIS3MX6* [pRS316-*SUS1*] [YGPM28j24] | This study |
| SY1006 | *MATa ura3∆0 leu2∆0 his3∆1 met15∆0 sus1∆::HIS3MX6* [pRS316-*SUS1*] [YGPM16d05] | This study |
| SY1007 | *MATa ura3∆0 leu2∆0 his3∆1 met15∆0 sus1∆::HIS3MX6* [pRS316-*SUS1*] [YGPM2m11] | This study |
| SY1008 | *MATa ura3∆0 leu2∆0 his3∆1 met15∆0 sus1∆::HIS3MX6* [pRS316-*SUS1*] [YGPM3j10] | This study |
| SY1009 | *MATa ura3∆0 leu2∆0 his3∆1 met15∆0 sus1∆::HIS3MX6* [pRS316-*SUS1*] [YGPM21b06] | This study |
| SY1010 | *MATa ura3∆0 leu2∆0 his3∆1 met15∆0 sus1∆::HIS3MX6* [pRS316-*SUS1*] [YGPM25c21] | This study |
| SY1011 | *MATa ura3∆0 leu2∆0 his3∆1 met15∆0 sus1∆::HIS3MX6* [pRS316-*SUS1*] [YGPM5f02] | This study |
| SY1012 | *MATa ura3∆0 leu2∆0 his3∆1 met15∆0 sus1∆::HIS3MX6* [pRS316-*SUS1*] [YGPM10e17] | This study |
| SY1013 | *MATa ura3∆0 leu2∆0 his3∆1 met15∆0 sus1∆::HIS3MX6* [pRS316-*SUS1*] [YGPM27n09] | This study |
| SY1014 | *MATa ura3∆0 leu2∆0 his3∆1 met15∆0 sus1∆::HIS3MX6* [pRS316-*SUS1*] [YGPM17n15] | This study |
| SY1015 | *MATa ura3∆0 leu2∆0 his3∆1 met15∆0 sus1∆::HIS3MX6* [pRS316-*SUS1*] [YGPM8d07] | This study |
| SY1016 | *MATa ura3∆0 leu2∆0 his3∆1 met15∆0 sus1∆::HIS3MX6* [pRS316-*SUS1*] [YGPM14m17] | This study |
| SY1017 | *MATa ura3∆0 leu2∆0 his3∆1 met15∆0 sus1∆::HIS3MX6* [pRS316-*SUS1*] [YGPM14h07] | This study |
| SY1018 | *MATa ura3∆0 leu2∆0 his3∆1 met15∆0 sus1∆::HIS3MX6* [pRS316-*SUS1*] [YGPM11b07] | This study |
| SY1019 | *MATa ura3∆0 leu2∆0 his3∆1 met15∆0 sus1∆::HIS3MX6* [pRS316-*SUS1*] [YGPM32o15] | This study |
| SY1020 | *MATa ura3∆0 leu2∆0 his3∆1 met15∆0 sus1∆::HIS3MX6* [pRS316-*SUS1*] [YGPM9h05] | This study |
| SY1021 | *MATa ura3∆0 leu2∆0 his3∆1 met15∆0 sus1∆::HIS3MX6* [pRS316-*SUS1*] [YGPM26i03] | This study |
| SY1022 | *MATa ura3∆0 leu2∆0 his3∆1 met15∆0 sus1∆::HIS3MX6* [pRS316-*SUS1*] [YGPM31j13] | This study |
| SY1023 | *MATa ura3∆0 leu2∆0 his3∆1 met15∆0 sus1∆::HIS3MX6* [pRS316-*SUS1*] [YGPM6e10] | This study |
| SY1024 | *MATa ura3∆0 leu2∆0 his3∆1 met15∆0 sus1∆::HIS3MX6* [pRS316-*SUS1*] [YGPM2p09] | This study |
| SY1025 | *MATa ura3∆0 leu2∆0 his3∆1 met15∆0 sus1∆::HIS3MX6* [pRS316-*SUS1*] [YGPM20m03] | This study |
| SY1026 | *MATa ura3∆0 leu2∆0 his3∆1 met15∆0 sus1∆::HIS3MX6* [pRS316-*SUS1*] [YGPM5d22] | This study |
| SY1027 | *MATa ura3∆0 leu2∆0 his3∆1 met15∆0 sus1∆::HIS3MX6* [pRS316-*SUS1*] [YGPM2h11] | This study |
| SY1031 | *MATa ura3∆0 leu2∆0 his3∆1 met15∆0 DBP5-GFP (S65T)::KlURA3* | This study |
| SY1032 | *MATa ura3∆0 leu2∆0 his3∆1 met15∆0 sus1∆::HIS3MX6* [pRS316] | This study |
| SY1033 | *MATa ura3∆0 leu2∆0 his3∆1 met15∆0 sus1∆::HIS3MX6* [pRS316-MEX67] | This study |
| SY1034 | *MATa ura3∆0 leu2∆0 his3∆1 met15∆0 sus1∆::HIS3MX6* [pRS316-DBP5] | This study |
| SY1035 | *MATa ura3∆0 leu2∆0 his3∆1 met15∆0 SUS1-HA::KlURA3* | This study |
| SY1036 | *MATa ura3∆0 leu2∆0 his3∆1 met15∆0 MEX67-GFP::HA-KlURA3* | This study |
| SY1037 | *MATa ura3∆0 leu2∆0 his3∆1 met15∆0 DBP5-GFP::HA-KlURA3* | This study |
| SY1038 | *MATa ura3∆0 leu2∆0 his3∆1 met15∆0* [pRS425] | This study |
| SY1039 | *MATa ura3∆0 leu2∆0 his3∆1 met15∆0 sus1∆::HIS3MX6* [pRS425] | This study |
| SY1040 | *MATa ura3∆0 leu2∆0 his3∆1 met15∆0 sus1∆::HIS3MX6* [pRS425-SUS1] | This study |
| SY1041 | *MATa ura3∆0 leu2∆0 his3∆1 met15∆0 sus1∆::HIS3MX6* [pRS425-MEX67] | This study |
| SY1042 | *MATa ura3∆0 leu2∆0 his3∆1 met15∆0 sus1∆::HIS3MX6* [pRS425-DBP5] | This study |
| SY1043 | *MATa ura3∆0 leu2∆0 his3∆1 met15∆0 sus1∆::HIS3MX6 DBP5-GFP (S65T)::KlURA3* | This study |
| SY1045 | *MATa ura3∆0 leu2∆0 his3∆1 met15∆0 sac3∆::KanMX4 DBP5-GFP (S65T)::KlURA3* | This study |
| SY1048 | *MATa ura3∆0 leu2∆0 his3∆1 met15∆0 sus1∆::HIS3MX6 sac3∆::KanMX4 DBP5-GFP (S65T)::KlURA3* | This study |
| SY1087 | *MATa ura3∆0 leu2∆0 his3∆1 met15∆0 sus1∆::HIS3MX6 thp1∆::KanMX4* | This study |
