# Supplementary material for: Sus1 maintains a normal lifespan through regulation of TREX-2 complex-mediated mRNA export
Source: Aging (Albany NY). 2022 Jun 29;14(12):4990–5012. doi: 10.18632/aging.204146 (PMC9271307; doi:10.18632/aging.204146)
Supplement: Supplementary Tables [file aging-14-204146-s003.pdf]

SUPPLEMENTARY TABLES

Supplementary Table 2. Plasmids used in this study.

Name	Description	Source
pFA6a-HIS3MX6	*pBR322 origin, Amp^R, HIS3MX6, HIS3 gene from S. kluyveri.*	[2]
pFA6a-KanMX6	*pBR322 origin, Amp^R, KanMX6, KanMX gene containins the known kanr ORF of the E. coli transposon Tn903.*	[2]
pFA6a-GFP-KlURA3	*pBR322 origin, Amp^R, URA3, GFP tag with URA3 gene*	[3]
pFA6a-HA-KlURA3	*pBR322 origin, Amp^R, URA3, Triple HA tag with URA3 gene*	[3]
pGP564	*2µ, Kan^R, LEU2*	[4]
YGPM2h11	*2µ, Kan^R, LEU2, [YBR109W-A]&, [ALG1], YSA1, SUS1, CYC8, YBR113W, RAD16, [LYS2]&*	[4]
YGPM5d22	*2µ, Kan^R, LEU2, [DAP1]&, MEX67, YPL168W, REV3, YPL166W, [SET6], [MLH3]&*	[4]
YGPM6e10	*2µ, Kan^R, LEU2, [CUE5]&, snR62, WHI2, YOR044W TOM6, DBP5, STD1, [RAT1]&*	[4]
YGPM25a15	*2µ, Kan^R, LEU2, [YKL187C] * MTR2 ASH1 SPE1 YKL183C-A [LOT5] **	[4]
YGPM17i23	*2µ, Kan^R, LEU2, [RSM23]* CWC23 SOH1 SCS3 MET13 MON1 RPS2 YGL123C-A NAB2 GPG1 [PRP43]*	[4]
pRS316	*CEN/ARS, Amp^R, URA3*	[5]
pRS316-SUS1	*CEN/ARS, Amp^R, URA3, SUS1 ORF with +- 900 bp of UTR*	This study
pRS316-MEX67	*CEN/ARS, Amp^R, URA3, MEX67 ORF with +- 900 bp of UTR*	This study
pRS316-DBP5	*CEN/ARS, Amp^R, URA3, DBP5 ORF with +- 900 bp of UTR*	This study
pRS425	*2µ, Amp^R, LEU2*	[6]
pRS425-SUS1	*2µ, Amp^R, LEU2, SUS1-HA with 900 bp upstream and 700 bp downstream of UTR*	This study
pRS425-MEX67	*2µ, Amp^R, LEU2, MEX67-HA with 900 bp upstream and 700 bp downstream of UTR*	This study
pRS425-DBP5	*2µ, Amp^R, LEU2, DBP5-HA with 900 bp upstream and 700 bp downstream of UTR*	This study

Supplementary Table 3. Mean lifespans and *P* values for RLS analysis.

Figure	Strain	Strain description	Mean lifespan	*P* value compared to matched control strain
Figure 1A	BY4741	WT	27.3	control
	SY495	*sus1Δ*	20.2	0.0006, ***
	SY652	*sus1Δ*	22.8	0.0120, *
	SY653	*sus1Δ*	19.0	0.0001, ***
	SY654	*sus1Δ*	20.8	0.0009, ***
Figure 1C	BY4741	WT	19.2	control
	SY495	*sus1Δ*	13.8	0.0131, *
	FY231	*ubp8Δ*	31.9	<0.0001, ***
	FY399	*sgf11Δ*	23.8	0.3267, ns
	FY402	*sgf73Δ*	45.4	<0.0001, ***
Figure 1D	BY4741	WT	24.2	control
	SY495	*sus1Δ*	18.9	0.0002, ***
	FY390	*thp1Δ*	7.2	<0.0001, ***

	FY391	*sac3Δ*	9.3	<0.0001, ***
	FY451	*sem1Δ*	22.5	0.0623, ns
Figure 2A	BY4741	WT	23.7	control
	SY495	*sus1Δ*	17.2	<0.0001, ***
	FY231	*ubp8Δ*	31.6	0.0020, **
	SY786	*sus1Δ ubp8Δ*	18.8	0.0014, **
Figure 2B	BY4741	WT	21.9	control
	SY495	*sus1Δ*	17.0	0.0051, **
	FY399	*sgf11Δ*	26.3	0.0291, *
	SY787	*sus1Δ sgf11Δ*	19.1	0.2784, ns
Figure 2C	BY4741	WT	23.5	Control
	SY495	*sus1Δ*	17.1	<0.0001, ***
	FY402	*sgf73Δ*	40.4	<0.0001, ***
	SY797	*sus1Δ sgf73Δ*	23.5	0.3870, ns
Figure 2D	BY4741	WT	21.8	control
	SY495	*sus1Δ*	16.7	0.0028, **
	FY391	*sac3Δ*	8.5	<0.0001, ***
	SY784	*sus1Δ sac3Δ*	12.5	<0.0001, ***
Figure 2E	BY4741	WT	25.6	control
	SY495	*sus1Δ*	16.6	<0.0001, ***
	FY390	*thp1Δ*	5.7	<0.0001, ***
	SY1087	*sus1Δ thp1Δ*	5.1	<0.0001, ***
Figure 2F	BY4741	WT	24.2	control
	SY495	*sus1Δ*	18.6	<0.0001, ***
	FY451	*sem1Δ*	20.6	0.0188, *
	SY785	*sus1Δ sem1Δ*	10.5	<0.0001, ***
Figure 3A	BY4741	WT	29.7	<0.0001, ***
	SY529	*sir2Δ*	12.4	control
	SY557	*sir2Δ ubp8Δ*	11.9	0.2598, ns
	SY559	*sir2Δ sgf11Δ*	13.9	0.1020, ns
	SY558	*sir2Δ sgf73Δ*	13.8	0.8582, ns
Figure 3B	BY4741	WT	29.3	<0.0001, ***
	SY529	*sir2Δ*	8.8	control
	SY653	*sus1Δ*	20.4	0.0006, ***
	SY699	*sir2Δ sus1Δ*	11.0	0.0200, *
Figure 3C	BY4741	WT	23.7	<0.0001, ***
	SY529	*sir2Δ*	13.2	control
	SY556	*sir2Δ thp1Δ*	5.5	<0.0001, ***
	SY532	*sir2Δ sac3Δ*	5.6	<0.0001, ***
Figure 4D	BY4741	WT	23.9	control
	FY395	*MEX67-DAmP*	18.3	0.0007, ***
	FY396	*MTR2-DAmP*	20.8	0.0781, ns

	SY971	*sus1Δ* + pGP564	9.5	control
Figure 4E	SY972	*sus1Δ* + YGPM2h11 (*SUS1*)	28.3	<0.0001, ***
	SY973	*sus1Δ* + YGPM5d22 (*MEX67*)	16.1	<0.0001, ***
	SY974	*sus1Δ* + YGPM6e10 (*DBP5*)	24.8	<0.0001, ***
	SY1039	*sus1Δ* + pRS425	18.6	control
Figure 4G	SY1040	*sus1Δ* + pRS425-*SUS1*	25.8	<0.0001, ***
	SY1041	*sus1Δ* + pRS425-*MEX67*	23.7	0.0023, **
	SY1042	*sus1Δ* + pRS425-*DBP5*	21.7	0.0408, *

***$P \leq 0.001$; **$P \leq 0.01$; *$P \leq 0.05$; ns, not significant.

SUPPLEMENTARY REFERENCES

1. Ryu HY, Ahn S. Yeast histone H3 lysine 4 demethylase Jhd2 regulates mitotic rDNA condensation. BMC Biol. 2014; 12:75. https://doi.org/10.1186/s12915-014-0075-3 PMID:25248920

2. Longtine MS, McKenzie A 3rd, Demarini DJ, Shah NG, Wach A, Brachat A, Philippsen P, Pringle JR. Additional modules for versatile and economical PCR-based gene deletion and modification in Saccharomyces cerevisiae. Yeast. 1998; 14:953–61. https://doi.org/10.1002/(SICI)1097-0061(199807)14:10<953::AID-YEA293>3.0.CO;2-U PMID:9717241

3. Sung MK, Ha CW, Huh WK. A vector system for efficient and economical switching of C-terminal epitope tags in Saccharomyces cerevisiae. Yeast. 2008; 25:301–11. https://doi.org/10.1002/yea.1588 PMID:18350525

4. Jones GM, Stalker J, Humphray S, West A, Cox T, Rogers J, Dunham I, Prelich G. A systematic library for comprehensive overexpression screens in Saccharomyces cerevisiae. Nat Methods. 2008; 5:239–41. https://doi.org/10.1038/nmeth.1181 PMID:18246075

5. Sikorski RS, Hieter P. A system of shuttle vectors and yeast host strains designed for efficient manipulation of DNA in Saccharomyces cerevisiae. Genetics. 1989; 122:19–27. https://doi.org/10.1093/genetics/122.1.19 PMID:2659436

6. Christianson TW, Sikorski RS, Dante M, Shero JH, Hieter P. Multifunctional yeast high-copy-number shuttle vectors. Gene. 1992; 110:119–22. https://doi.org/10.1016/0378-1119(92)90454-w PMID:1544568
